# Combined treatment with amitraz and thymol to manage *Varroa destructor* mites (Acari: Varroidae) in *Apis mellifera* honey bee colonies (Hymenoptera: Apidae)

**DOI:** 10.1093/jisesa/ieae022

**Published:** 2024-05-28

**Authors:** Dan Aurell, Clint Wall, Selina Bruckner, Geoffrey R Williams

**Affiliations:** Department of Entomology and Plant Pathology, Auburn University, Auburn, AL 36849, USA; Department of Entomology and Plant Pathology, Auburn University, Auburn, AL 36849, USA; Department of Entomology and Plant Pathology, Auburn University, Auburn, AL 36849, USA; Department of Entomology and Plant Pathology, Auburn University, Auburn, AL 36849, USA

**Keywords:** integrated pest management, cultural control, chemical control, amitraz, thymol

## Abstract

The parasitic mite *Varroa destructor* (Anderson and Trueman) is one of the greatest stressors of *Apis mellifera* (L.) honey bee colonies. When *Varroa* infestations reach damaging levels during fall, rapid control is necessary to minimize damage to colonies. We performed a field trial in the US Southeast to determine if a combination of registered treatments (Apivar, amitraz-based; and Apiguard, thymol-based) could provide rapid and effective control of *Varroa*. We compared colonies that received this combination treatment against colonies that received amitraz-based positive control treatments: (i) Apivar alone; or (ii) amitraz emulsifiable concentrate (“amitraz EC”). While not registered, amitraz EC is used by beekeepers in the United States in part because it is thought to control *Varroa* more rapidly and effectively than registered products. Based on measurements of *Varroa* infestation rates of colonies after 21 days of treatment, we found that the combination treatment controlled *Varroa* nearly as rapidly as the amitraz EC treatment: this or other combinations could be useful for *Varroa* management. At the end of the 42-day trial, colonies in the amitraz EC group had higher bee populations than those in the Apivar group, which suggests that rapid control helps reduce *Varroa* damage. Colonies in the combination group had lower bee populations than those in the amitraz EC group, which indicates that the combination treatment needs to be optimized to avoid damage to colonies.

## Introduction

*Varroa destructor* (hereafter “*Varroa*”) is a parasitic mite which seriously damages colonies of *Apis mellifera* (hereafter “honey bee” or “bee”), particularly in temperate regions. Along with *Varroa*-transmitted viruses, it compromises individual honey bees and whole colonies, which reduces productivity and increases colony mortality (Currie 2008, Genersch et al. 2010, Traynor et al. 2020). Without adequate management, *Varroa* can cause severe losses of honey bee colonies (Guzmán-Novoa et al. 2010), and US beekeepers consider *Varroa* the leading cause of winter losses (Aurell et al. 2024a, Bruckner et al. 2023). Although *Varroa* causes loss of colonies over winter, much of the damage occurs earlier: in late summer and fall. *Varroa* infestation rates of colonies often peak at this time (Traynor et al. 2016) as a result of natural decreases in the bee population and continued increases in the *Varroa* population of the colonies (Martin 1998). During fall, infestation rates often rise far above the commonly cited treatment threshold of 3% (i.e., 3 *Varroa* per 100 adult bees; Traynor et al. 2016). This is also when long-lived winter bees are produced, which are critical for colony survival over winter (Amdam et al. 2004). To prevent damage to these winter bees, it is important to use treatments that reduce infestation rates in late summer and fall before these winter bees develop (Frey and Rosenkranz 2014), which would be best accomplished with treatments that act rapidly. The idea of a “rapid treatment” is relative, but since worker bees take 21 days to develop into adults, we suggest that a treatment that controls *Varroa* populations in 21 days instead of 42 days could meaningfully benefit colony health.

*Varroa* mites occur in 2 locations in the hive: on the adult bees and in wax-capped cells that contain developing bees (hereafter “brood”). These are termed the dispersal and the reproductive phases, respectively (Traynor et al. 2020). Under normal conditions when brood is present in a honey bee colony, some *Varroa* will be in the dispersal phase and some will be in the reproductive phase. The presence of *Varroa* in the reproductive phase poses challenges for Integrated Pest Management (IPM)—both in terms of determining economic thresholds for *Varroa* and in terms of developing control methods to reduce infestations when they reach damaging levels (Jack and Ellis 2021). When *Varroa* are infesting capped brood cells, they are relatively protected from most chemical treatments that are available because the brood cells are closed with a wax capping (e.g., Koeniger and Fuchs 1988, Berry et al. 2021).

Chemical control is the most effective way to reduce *Varroa* infestation rates (Jack and Ellis 2021), and a majority of both small-scale and large-scale beekeepers use Varroacides based on naturally occurring or synthetic chemicals (Haber et al. 2019). Natural compounds widely used as Varroacides include thymol, oxalic acid, and formic acid (Haber et al. 2019), but their safety for colonies and efficacy against *Varroa* depends on weather and colony conditions (including the presence of capped brood). In contrast, a widely used synthetic Varroacide—amitraz—is effective in a wide range of conditions; hence, many large-scale beekeepers in the United States use amitraz (Haber et al. 2019). The US-registered formulation Apivar releases amitraz slowly from plastic strips installed in the hive (Floris et al. 2001) which causes elevated *Varroa* mortality for several weeks during the treatment period of 42–56 days (Vandervalk et al. 2014). However, it is not recommended when infestation rates are already at damaging levels (Honey Bee Health Coalition 2021). Some beekeepers also resort to using emulsifiable concentrate formulations of amitraz (hereafter “amitraz EC”; active ingredient, amitraz 12.5%), despite these formulations not being registered for use on honey bees in the United States (Honey Bee Health Coalition 2021, Jack and Ellis 2021). This is in part because beekeepers anecdotally report more rapid and effective control with amitraz EC than with Apivar, which is consistent with recent findings (Jack et al. 2024) and in part because the cost per treatment is low (Honey Bee Health Coalition 2021). To address unregistered product use and the emergence of amitraz-resistant *Varroa* (Rinkevich 2020), it is pressing to develop registered treatment options that do not rely solely on amitraz, but still control *Varroa* effectively and rapidly, while being safe for colonies.

Combining treatments has been suggested as a potential avenue to improve *Varroa* IPM (Jack and Ellis 2021), and combinations involving amitraz have been tested against other arachnids such as the tick *Rhipicephalus microplus* (Rodriguez-Vivas et al. 2018). A combination of registered products could potentially be used to match the rapid and effective *Varroa* control that beekeepers report from amitraz EC treatment. However, only a few investigators have tested combinations of chemical Varroacides (e.g., Toomemaa 2019, Pietropaoli and Formato 2022).

We decided to test a combination of Apivar (active ingredient, amitraz 3.3%) and Apiguard (active ingredient, thymol 25%) as there is no synergistic toxicity to adult honey bees between thymol and amitraz (Johnson et al. 2013). Because Apivar is one of the most effective registered treatments when brood is present (Honey Bee Health Coalition 2022), we used this as the foundation of the combination. We added Apiguard because this treatment can be used up to daytime maximum temperatures of 40.6 °C (105 °F) (US EPA 2018), can be effective even in the presence of brood (Giacomelli et al. 2016), and because we are not aware of reports of *Varroa* resistance to thymol (Jack and Ellis 2021). Apiguard is known to cause some brood reduction (Floris et al. 2004), which would force more of the *Varroa* population into the dispersal phase on adult honey bees where they are more vulnerable to treatments. Thus, we intended to use the chemical treatment Apiguard to induce cultural control of *Varroa* through brood suppression—in addition to providing direct chemical control.

The purpose of this study was to test the performance of a combined Apivar and Apiguard against the performance of 2 amitraz-based treatments (Apivar; amitraz EC). For the purposes of this study, these amitraz-based treatments are considered positive controls or “standard of care” treatments against *Varroa* because nearly all large-scale beekeepers apply Varroacides in their operations (Haber et al. 2019). We hypothesized that a combination treatment of Apivar and Apiguard would control *Varroa* more rapidly and effectively than Apivar alone—and equally well as amitraz EC. To test this hypothesis, we performed a field trial in which colonies received either: (i) Apivar; (ii) amitraz EC; or (iii) a combination of Apivar and Apiguard. To assess the ability of these treatments to control *Varroa* rapidly and effectively, and to assess their effects on colonies, we monitored *Varroa* infestation rates and colony strength parameters before, during, and after treatment. To determine whether “rapid” control of *Varroa* was achieved, we assessed *Varroa* infestations on day 21, and to determine the overall relative “effectiveness” of *Varroa* control, we assessed *Varroa* infestations on day 42.

## Materials and Methods

### Overview

For this trial, all honey bee colonies were located in 1 experimental apiary in Auburn, AL, USA (32.6266°N, 85.5443°W). On September 23rd, 2020 (hereafter “day 0”), we applied 1 of 3 treatments to each colony: (i) Apivar; (ii) amitraz EC; or (iii) a combination of Apivar and Apiguard. Major data collection events occurred on 3 occasions: pretreatment, mid-treatment, and post-treatment. We began these assessments 4–5 days before treatment, on day 21, and on day 42, respectively (Supplementary Fig. S1). We hereafter refer to these data collection points as day 0, 21, and 42. On all 3 occasions, we assessed: adult bee population; brood population; and *Varroa* infestation rates of adult bees and of brood. According to nearby weather station data, daily high temperatures ranged from 15 to 29 °C, and daily low temperatures ranged from 1 to 21 °C between day 0 and 42 (NOAA-NCEI 2023).

### Colony Preparation and Management

We selected 47 trial colonies from a prepared pool of approximately 60 colonies headed by first-year queens of European-type stock (i.e., non-Africanized). To equalize the strength of this pool of colonies and to reduce variation in *Varroa* infestation rates, we moved frames of capped brood between colonies but finished this intervention at least 4 wk before the beginning of the trial to allow transient effects (Fox and Gurevitch 2000) on the *Varroa* population to stabilize. Due to inclusion in another trial, some colonies had been subjected to other treatments before the start of this trial, but we think the previous treatments are unlikely to have influenced the results of the present trial. Specifically, 7 experimental colonies had previously been treated with 2 applications of oxalic acid vapor, ending 4 or more weeks before the trial started. This exceeds the developmental time of bees and the length of the reproductive cycle of *Varroa* in the presence of brood (Boot et al. 1994), and should have allowed any transient effects on *Varroa* populations to dissipate. Another 7 experimental colonies had been treated with oxalic acid vapor 7 times, ending 1 wk before the trial started. As a precaution, we assigned these latter colonies as evenly as possible to treatment groups. Further supporting the likely low impact of these previous oxalic acid treatments, applications were made according to the label at a rate of 1g per brood chamber, which prevents *Varroa* infestations from increasing when brood is present but does not reduce infestations (Berry et al. 2021). Each hive consisted of 2 deep 10-frame Langstroth brood chambers (comb area 880 cm^2^ per side of each frame; Delaplane et al. 2013) on screened bottom boards with 3.2 mm (1/8″) steel mesh. The bottom brood chamber of each hive contained 9 frames; the top brood chamber contained 8 frames and a 5.7 L (1.5 gallon) frame feeder. To replicate an entrance size typical of commercial hives in the United States, we used entrance reducers to restrict each entrance to 18 cm^2^. We selected 47 colonies from this cohort based on pretreatment assessment, excluding colonies that were outliers on *Varroa* infestation rate or colony strength, were not queenright, or that showed visual signs of European Foulbrood (“EFB”; *n* = 2).

We treated all colonies with 200 mg of oxytetracycline in powdered sugar on day 0, 7, and 14 according to the label to control EFB and fed 6.2 L of sucrose syrup (50% w/v) to each colony between day 8 and day 34. We kept the screened bottom boards on all hives tightly closed with a plastic slide-in insert at the beginning of the trial to moderate the release of thymol vapors from the hives treated with Apiguard. We checked the queen status of colonies approximately weekly during the experiment (Supplementary Fig. S1) by confirming the presence of eggs and optionally by locating the queen visually.

### Assessment of *Varroa* Infestation Rate

To determine *Varroa* infestation rates of adult honey bees, we performed a triple rinse alcohol wash on a sample of approximately 300 honey bees (Dietemann et al. 2013). First, we collected adult bees from one or more brood frames that contained older open larvae by shaking the frame over a plastic basin, allowing bees to fly away for approximately 10 s, and transferring a 118-ml (i.e., 1/2 cup) sample of bees into a jar containing enough 35% isopropyl alcohol solution to submerge the bees. Then, we added more 35% isopropyl alcohol to the jar so that it contained at least 500 ml and shook the jar manually for 1 min. Then, we rinsed each sample using the isopropyl alcohol solution through a pair of sieves: a coarse mesh sieve to catch bees but allow *Varroa* to pass through, and a fine mesh sieve to catch *Varroa*. Then, we repeated the rinse procedure 2 more times, shaking the coarse sieve between rinses to turn the bees. After 3 rinses, we strained the solution using the fine sieve and counted the number of *Varroa* recovered. To validate this method (Dietemann et al. 2013), we also continued rinsing some samples (*n* = 115) until 2 *Varroa*-free rinses were achieved. Of 2,343 total *Varroa* collected using this more thorough protocol, 2,313 (98.7%) had already been retrieved after 3 rinses, which indicates a high recovery rate of the triple rinse method. For analyses, we used *Varroa* counts from the triple rinse procedure to ensure a consistent effort across samples. We weighed the entire sample of honey bees and separately weighed 100 bees from the sample, which allowed us to calculate an estimated number of bees per sample (Eq. 1) (mean = 321.7; min = 251.4; max = 412.5; sd = 39.1).


$$\begin{array}{l} Number\;of\;bees\;in\;sample=\;100\times \;\frac{Weight\;of\;all\;bees}{Weight\;of\;100\;bees} \end{array}$$ (1)

Based on the number of *Varroa* recovered and the estimated number of bees per sample, we calculated the number of *Varroa* per 100 adult bees and expressed this as a % infestation rate (i.e., 1% = 1 *Varroa* per 100 bees).

To assess the *Varroa* infestation rate of brood, we selected 2 frames well occupied with capped brood from each colony (Branco et al. 2006) and uncapped cells to determine whether they were infested by *Varroa* mites or not. Per colony, we assessed the infestation rate of 200 capped worker brood cells, across 4 distinct areas with 50 cells each when possible. We uncapped cells in a linear pattern to assess infestation across a range of developmental stages. Using fine-tipped forceps and a light source, we opened each cell and inspected the cell capping, the brood, and the interior of the cell. We scored the cell as infested if adults, offspring, or fecal deposits of *Varroa* were observed. During the pretreatment and day 42 assessments, we assessed the *Varroa* infestation rate of brood in all colonies; due to the time required for brood examinations, we collected these data for 35 colonies on day 21.

### Assessment of Colony Strength

We assessed the strength of colonies using the “subjective mode” to estimate visually the percent coverage of resources on frames (Delaplane et al. 2013, Dainat et al. 2020). For both sides of every frame in each colony, we estimated the population of adult bees, capped worker brood cells (developing workers), and capped drone brood cells. To obtain a colony-level estimate for each variable, we summed the percent coverage across all frames in the colony and divided by 2. Therefore, a “frame of bees” or “frame of brood” refers to a deep Langstroth frame completely covered on both sides with that resource. Whenever a colony contained less than 1.25 frames fully occupied with adult bees, it was removed from the apiary and the experiment to minimize the chance of *Varroa* transmission through robbing of failing colonies (Peck and Seeley 2019).

### Varroacide Treatments

After pretreatment assessments, we randomly assigned colonies to 1 of 3 treatment groups while attempting to keep key variables homogeneous across groups. We re-randomized group assignments until we obtained 1 that provided homogeneity of key variables (number of adult bees, % *Varroa* infestation rate of adult bees, and number of capped worker brood cells) across groups, good spatial interspersion of treatments (Hurlbert 1984), and a good representation of colonies with all treatment histories in all groups. The 3 treatment groups were: (i) Apivar; (ii) amitraz EC; and (iii) combination.

In the Apivar group (16 colonies), 2 strips of Apivar (Veto-Pharma, Palaiseau, France) were applied in each brood chamber (4 strips total per colony). The strips were positioned centrally in the area of adult bee activity and brood with at least 2 frames between strips, according to the label (US EPA 2021). The strips were suspended with a toothpick to allow bees to contact both sides of the strips and to avoid brood damage. The Apivar strips were installed on day 0 and removed on day 42—the default maximum treatment time according to the US label.

In the amitraz EC group (15 colonies), we applied Scott brand shop towels (1/2 sheet, 14.6 cm × 27.0 cm) between the brood chambers (i.e., flat across the top bars of the frames in the bottom box, centrally, and perpendicular to the top bars). Each towel contained 12 ml of a 1:4 mixture by volume of amitraz EC:canola oil. Anecdotally, this is a typical treatment method. We applied a newly prepared towel on day 0, 7, and 14, after removing any previous towel, and removed the third towel on day 21. We expected the original amitraz EC concentrate to contain approximately 12.5% amitraz (w/v), and thus expected the mixture in the prepared towels to contain approximately 2.5% amitraz (w/v). To confirm this, we sent *n* = 9 samples of the prepared towels for analysis by gas chromatograph coupled with mass spectrometry (GC/MS) (EPA, Athens, GA, USA). There, the oil-concentrate mixture was removed from the towels via centrifugation and approximately 5 mg was transferred to a 15 ml centrifuge tube. Next, 10 ml acetonitrile was added and the mixture vortexed and gently shaken overnight. Two microliters were then transferred to GC/MS vials and diluted in dichlormethane. Amitraz and its metabolites N-(2,4-dimethylphenyl)-N-methylformamidine and 2,4-dimethylaniline were analyzed in selected ion monitoring mode. The mixtures from the prepared towels were found to contain a mean of 2.47% amitraz (min = 2.34; max = 2.77) with minimal detection of metabolites. We applied the amitraz EC treatment under an Experimental Use Permit from the Alabama Department of Agriculture & Industries (permit number, 2020-EUP-1).

In the combination group (16 colonies), colonies were treated with Apivar according to the label (as in the Apivar group) and also received a single dose of 51 ml of Apiguard gel (Vita-Europe Ltd., Basingstoke, Hants, United Kingdom). The gel was applied to a dosing card which was placed on the center of the top bars of the frames of the bottom box. We made only 1 Apiguard application as opposed to the 2 specified on the label (US EPA 2018) because the product can result in brood reduction (Floris et al. 2004) and we did not want to cause excessive brood reduction and thus endanger the production of winter bees that are essential for colony survival over winter (Amdam et al. 2004). The Apivar strips and Apiguard gel were applied at the same time on day 0; Apivar strips and any dosing card remnants were removed on day 42.

Because the high starting *Varroa* infestation rates of colonies strongly indicated the need for treatment, we compared the combination treatment against the 2 positive control treatments (Apivar and amitraz EC). We omitted an untreated negative control group to maintain the integrity of the experiment, because untreated colonies would likely have rapidly weakened or collapsed, leading to robbing and excessive spread of *Varroa* from untreated to treated colonies (Dietemann et al. 2013). To test whether the addition of Apiguard could result in superior *Varroa* control over Apivar alone, an Apiguard-only treatment was not required, and to ensure sufficient sample size per group, we did not include an Apiguard-only treatment.

### Statistical Analysis

We analyzed and visualized the data using R 4.2.2 (R Core Team 2022) and the *tidyverse* tools (Wickham et al. 2019). We used the *dplyr* package for data preparation (Wickham et al. 2023), the *lme4* package for model fitting (Bates et al. 2015), the *emmeans* package for interpretation of model results (Lenth 2020), the *ggplot2* package for data visualization (Wickham 2016), and the *gt* package for generating tables (Iannone et al. 2022). We used an alpha level of 0.05 for significance tests.

To prepare the data, we first excluded data from 6 colonies that experienced queen loss between day 0 and day 42—because queen loss disrupts the population dynamics of both honey bees and *Varroa* in a colony. Therefore, our analytical dataset contained 41 colonies. We retained in the data set the colonies that were removed before the end of the trial (*n* = 4) and that died (*n* = 1), despite the missing data for these colonies at later assessment times. We also used the *floor* function to transform the estimated number of bees in a sample of adult bees to the next lowest integer.

We fitted statistical models for 4 response variables of interest, fitting a priori models of the form *response ~ treatment + day + treatment × day* (Table 1). The experimental intervention justified estimating the effect of each predictor, so we retained each of these model terms whether they were statistically significant or not. To determine the significance of these effects, we compared the a priori model against nested reduced models with an analysis of deviance (using the *anova* function). This is analogous to the process of simplifying models by stepwise deletion (Crawley 2013) except that all terms in the a priori models were retained. We also explored the possibility that starting *Varroa* infestation rates could influence the colony strength variables (frames of adult bees, frames of worker brood) because *Varroa* infestation weakens colonies. We therefore considered including the pretreatment *Varroa* infestation rate of adult bees as a covariate for colony strength variables, as well as the interaction of pretreatment *Varroa* infestation rate × experiment day (Table 1)—but we only kept these effects in the final model when they significantly improved model fit during forward model selection (Crawley 2013). By controlling for effects of *Varroa* parasitism, we hoped to reduce swamping and improve our ability to detect treatment effects on colony strength.

**Table 1.** Statistical models used to analyze data on *Varroa destructor* infestation rates of *Apis mellifera* honey bee colonies, the population of *V. destructor* in capped worker brood cells, and the strength of *A. mellifera* colonies. Models 1, 2, 4, and 6 were the final models used to report results, as indicated by bold font. Models were fitted using the *lme4* package in R 4.2.2. All models also included a random intercept effect of colony ID (not shown)

No.	Response variable description	Model formula^a^	Model type
1	Number of *Varroa* in sample of adult bees	**Varroa ~ trt + day + trt:day + offset(log(bees_in_sample))**	GLMM, Neg. binomial (log link)
2	Number of infested worker cells (in 200 worker cells)	**infested ~ trt + day + trt:day**	GLMM, Neg. binomial (log link)
3	Frames of adult bees	frames ~ trt + day + trt:day	LMM, Normal
4	Frames of adult bees	**frames ~ trt + day + trt:day + pre_Varroa + pre_Varroa:day**	LMM, Normal
5	Frames of capped worker brood	frames ~ trt + day + trt:day	LMM, Normal
6	Frames of capped worker brood	**frames ~ trt + day + trt:day + pre_Varroa**	LMM, Normal

trt = “treatment” (categorical); day = “experiment day” (categorical); bees_in_sample = “number of bees in sample” (integer); pre_Varroa = “*Varroa* per 100 bees at pretreatment assessment” (continuous).

^a^Model formulas are written in the form required by the *lme4* package (Bates et al. 2015).

We analyzed experiment day as a categorical predictor to allow nonlinear relationships over time. We fitted all models as mixed-effects models with colony ID as a random effect with a random intercept to avoid pseudoreplication in the repeated measures design (Hurlbert 1984). We fit these as linear mixed models (LMMs) or generalized linear mixed models (GLMMs) as appropriate to the characteristics of the data. We performed visual inspection of the plotted residuals of the final models for the colony strength variables and did not observe major violations of normality. For the *Varroa* infestation rate of adult bees, the *Varroa* infestation rate of worker brood, and the population of *Varroa* in worker brood cells, we fitted GLMMs with a negative binomial distribution instead of a Poisson distribution because the variance-to-mean ratios of 22.5, 14.2, and 5.2, respectively, indicated that Poisson models would be inappropriate due to overdispersion. For *Varroa* infestation rate of adult bees, we also included an offset in the model to account for the differing numbers of bees in each sample. For the models with a normal distribution we compared models fitted by maximum likelihood but obtained results from models fitted by REML. For the models with a negative binomial distribution we used only models fitted by maximum likelihood because REML models were not available in the package.

From each final model, we generated point estimates and 95% confidence intervals (CIs) using the *emmeans* package, and saved these for plotting with *ggplot2* and table generation with *gt*. When there was a significant interaction of *treatment* × *day*, we used the *joint_test* function in the *emmeans* package to test whether there were differences between treatment groups within each day. When there were, we then used the *emmeans* package to perform post hoc tests between the 3 groups within each date using the Tukey multiplicity adjustment for *P*-values. For normally distributed models, we used the Kenward–Roger method of determining degrees of freedom, but for the negative binomial-distributed models, we used the *t*-as-*z* method and therefore report *df* = ∞ (Luke 2017, Lenth 2020).

## Results

### Effects of Treatment on *Varroa* Infestation Rate of Adult Bees

**Fig. 1.**
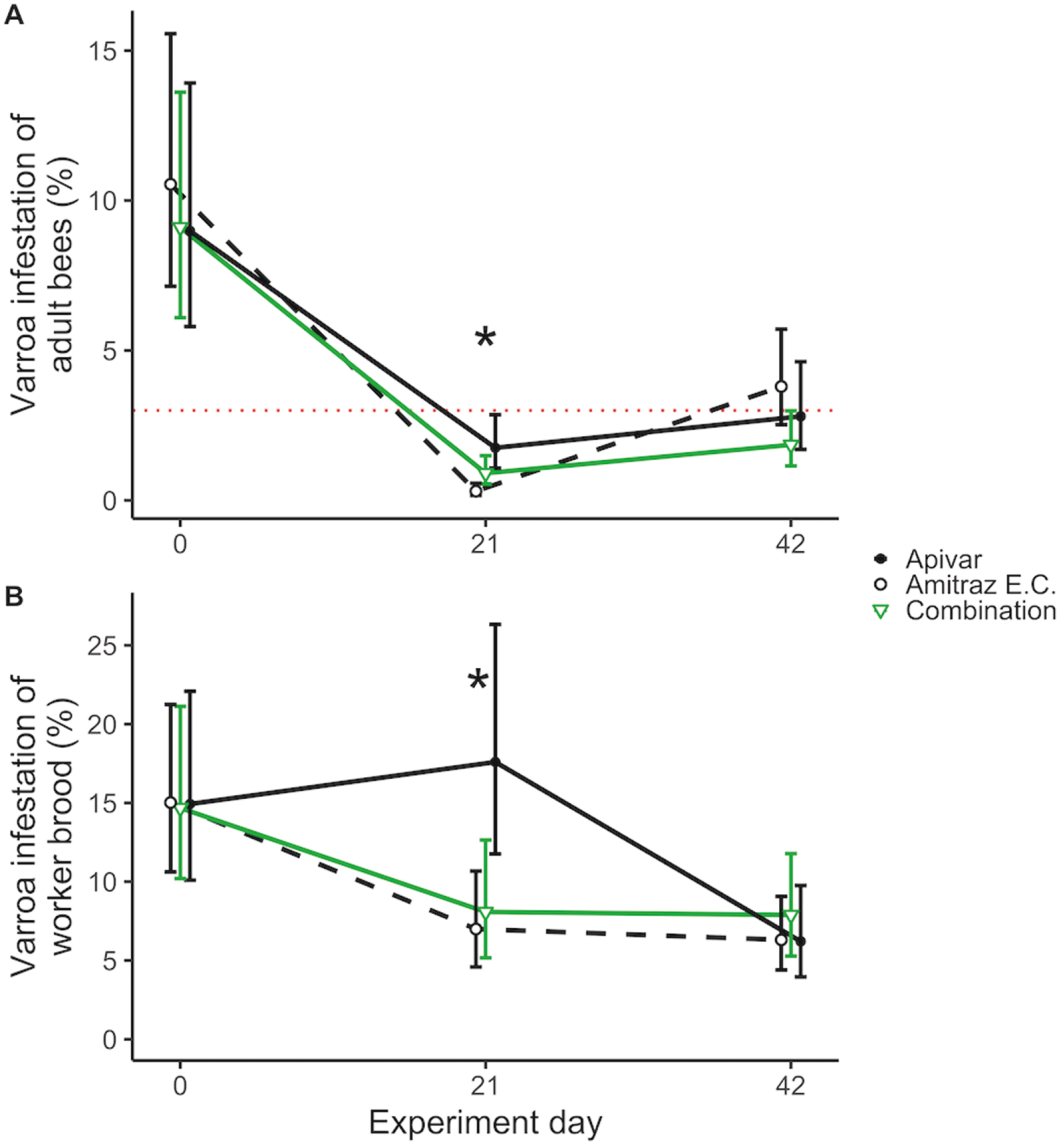
*Varroa destructor* infestation rates of *Apis mellifera* honey bee colonies. A) Percent *Varroa* infestation of adult bees. The horizontal dotted line represents 3% infestation of adult bees, above which treatment is commonly recommended. B) Percent *Varroa* infestation of capped worker brood cells. The dotted line is omitted for brood infestation because there is no commonly used threshold for brood infestation. Estimated marginal means within days are shown based on generalized linear mixed models no. 1 and 2, respectively (Table 1), and error bars indicate 95% confidence intervals. Asterisks indicate 1 or more pairwise differences between treatments within that day significant at *P* < 0.05. Point estimates and 95% confidence intervals can be found in Table 2.

We found a significant interaction of treatment × experiment day on *Varroa* infestation rate of adult bees (χ^2^_*df* = 4_ = 26.733, *P* < 0.001). On day 0, we found no significant differences between the *Varroa* infestation rate of adult bees in any groups (*F* ratio_2,∞_ = 0.187, *P* = 0.829), with estimated *Varroa* infestation rates 9.0%, 10.5%, and 9.1% for Apivar, amitraz EC, and combination groups, respectively (Fig. 1; estimates and 95% CIs in Table 2)—all substantially above the commonly cited threshold of 3%. On day 21, there were significant differences between treatments (*F* ratio_2,∞_ = 9.39, *P* < 0.001). Adult bees in the amitraz EC group had an estimated *Varroa* infestation rate of 0.3%, whereas the Apivar and combination group had 1.8% and 0.9%, respectively. *Varroa* infestation rates in the Apivar group were significantly higher than in the amitraz EC group (*z*-ratio_∞_ = 4.33, *P* < 0.001); *Varroa* infestation rates in the combination group were also significantly higher than in the amitraz EC group (*z*-ratio_∞_ = 2.67, *P* = 0.021). While we estimated that the infestation rate of the Apivar group was twice that of the combination group, this difference was not statistically significant (*z*-ratio_∞_ = 1.87, *P* = 0.148) and no groups were above the threshold. On day 42, though we did not find significant differences between groups (*F* ratio_2,∞_ = 2.54, *P* = 0.079), the estimated infestation rates of the Apivar, amitraz EC and combination groups were 2.8%, 3.8%, and 1.9%, respectively. On day 42, the amitraz EC group was the only group whose estimated mean infestation level was above the threshold.

**Table 2.** Point estimates and 95% confidence intervals for *Varroa destructor* infestation variables and *Apis mellifera* honey bee colony strength variables. Point estimates are estimated marginal means predictions from fitted statistical models (Table 1). Models were fitted using the *lme4* package and predictions were obtained using the *emmeans* package in R 4.2.2

Variable	Experiment day	Treatment	*n*	Estimate [95% CI]
% Varroa infestation (adult bees)	0	Apivar	12	8.99 [5.80–13.92]
	0	Amitraz EC	15	10.54 [7.14–15.56]
	0	Combination	14	9.11 [6.09–13.61]
	21	Apivar	12	1.75 [1.08–2.86]
	21	Amitraz EC	15	0.31 [0.16–0.57]
	21	Combination	14	0.91 [0.55–1.49]
	42	Apivar	10	2.80 [1.70–4.62]
	42	Amitraz EC	15	3.80 [2.53–5.71]
	42	Combination	12	1.85 [1.15–2.99]
% Varroa infestation (capped worker brood)	0	Apivar	12	14.93 [10.09–22.08]
	0	Amitraz EC	15	15.02 [10.62–21.25]
	0	Combination	14	14.68 [10.20–21.13]
	21	Apivar	11	17.60 [11.76–26.32]
	21	Amitraz EC	11	7.00 [4.59–10.67]
	21	Combination	10	8.08 [5.16–12.65]
	42	Apivar	10	6.21 [3.95–9.76]
	42	Amitraz EC	15	6.31 [4.39–9.06]
	42	Combination	12	7.89 [5.28–11.79]
Frames of bees	0	Apivar	12	4.01 [3.22–4.80]
	0	Amitraz EC	15	4.31 [3.60–5.01]
	0	Combination	14	4.43 [3.70–5.16]
	21	Apivar	12	3.27 [2.48–4.06]
	21	Amitraz EC	15	4.37 [3.66–5.07]
	21	Combination	14	2.76 [2.03–3.49]
	42	Apivar	10	2.91 [2.07–3.75]
	42	Amitraz EC	15	4.30 [3.60–5.01]
	42	Combination	12	2.94 [2.17–3.71]
Frames of capped worker brood	0	Apivar	12	2.41 [2.14–2.69]
	0	Amitraz EC	15	2.68 [2.43–2.92]
	0	Combination	14	2.67 [2.42–2.93]
	10	Apivar	12	1.13 [0.85–1.40]
	10	Amitraz EC	15	1.22 [0.97–1.46]
	10	Combination	14	0.73 [0.48–0.99]
	21	Apivar	12	1.23 [0.96–1.51]
	21	Amitraz EC	15	1.40 [1.15–1.65]
	21	Combination	14	1.33 [1.08–1.59]
	42	Apivar	10	0.66 [0.37–0.96]
	42	Amitraz EC	15	0.84 [0.60–1.09]
	42	Combination	12	0.60 [0.33–0.87]

## Effects of Treatment on *Varroa* Infestation Rates of Capped Worker Brood

We found a significant interaction of treatment × experiment day on infestation rate of capped worker brood (χ^2^_4_ = 9.74, *P* = 0.045). On day 0, we found no significant differences between the *Varroa* infestation rate of capped worker brood in the Apivar, amitraz EC, and combination groups (*F* ratio_2,∞_ = 0.004, *P* = 0.996), with estimated infestation rates of 14.9%, 15.0%, and 14.7%, respectively (Fig. 1; estimates and 95% CIs in Table 2). On day 21, there were significant differences between groups (*F* ratio_2,∞_=5.68, *P* = 0.003). Our estimate of *Varroa* infestation rate of brood in the Apivar group remained high (17.6%), while the infestation rates in the amitraz EC and combination groups were 7.0% and 8.1%, respectively. On day 21, we observed significantly higher rates of brood infestation in colonies treated with Apivar than those treated with amitraz EC (*z*-ratio_∞_ = 3.12, *P* = 0.005) or the combination treatment (*z*-ratio_∞_ = 2.57, *P* = 0.027), which did not differ significantly from each other (*z*-ratio_∞_ = 0.465, *P* = 0.888). On day 42, the infestation rates of brood were not significantly different between groups (*F* ratio_2,∞_ = 0.427, *P* = 0.653), with estimated 6.2%, 6.3%, and 7.9% infestation rates of worker brood in the Apivar, amitraz EC, and combination groups, respectively.

## Effects of Treatment on Adult Bee Population

The interaction of treatment × experiment day on adult bee population was significant (χ^2^_4_ = 12.10, *P* = 0.017). When analyzing the effect of treatment on adult bee population, the model fit was significantly improved by including the effect of day-0 infestation rate on bee population (χ^2^_1_ = 25.04, *P* < 0.001). The interaction of day-0 infestation rate × experiment day on bee population also significantly improved model fit (χ^2^_2_ = 7.88, *P* = 0.020). For each additional 1% *Varroa* infestation of adult bees on day 0, we found that colonies had 0.10 fewer frames of bees (95% CI: 0.04–0.16) on that day. Colonies with higher *Varroa* infestation rates on day 0 also suffered further reductions in bee population through the trial: For each additional 1% *Varroa* infestation of adult bees on day 0, we observed that colonies also had 0.08 fewer frames of bees (95% CI: 0.02–0.14) on day 21 and 0.08 fewer frames of bees (95% CI: 0.01–0.14) on day 42. Relative to a colony with a 0% infestation rate, a colony with a 10% infestation rate would therefore have a deficit of 0.8 frames of bees on day 0, and a deficit of 1.6 frames of bees on days 21 and 42.

**Fig. 2.**
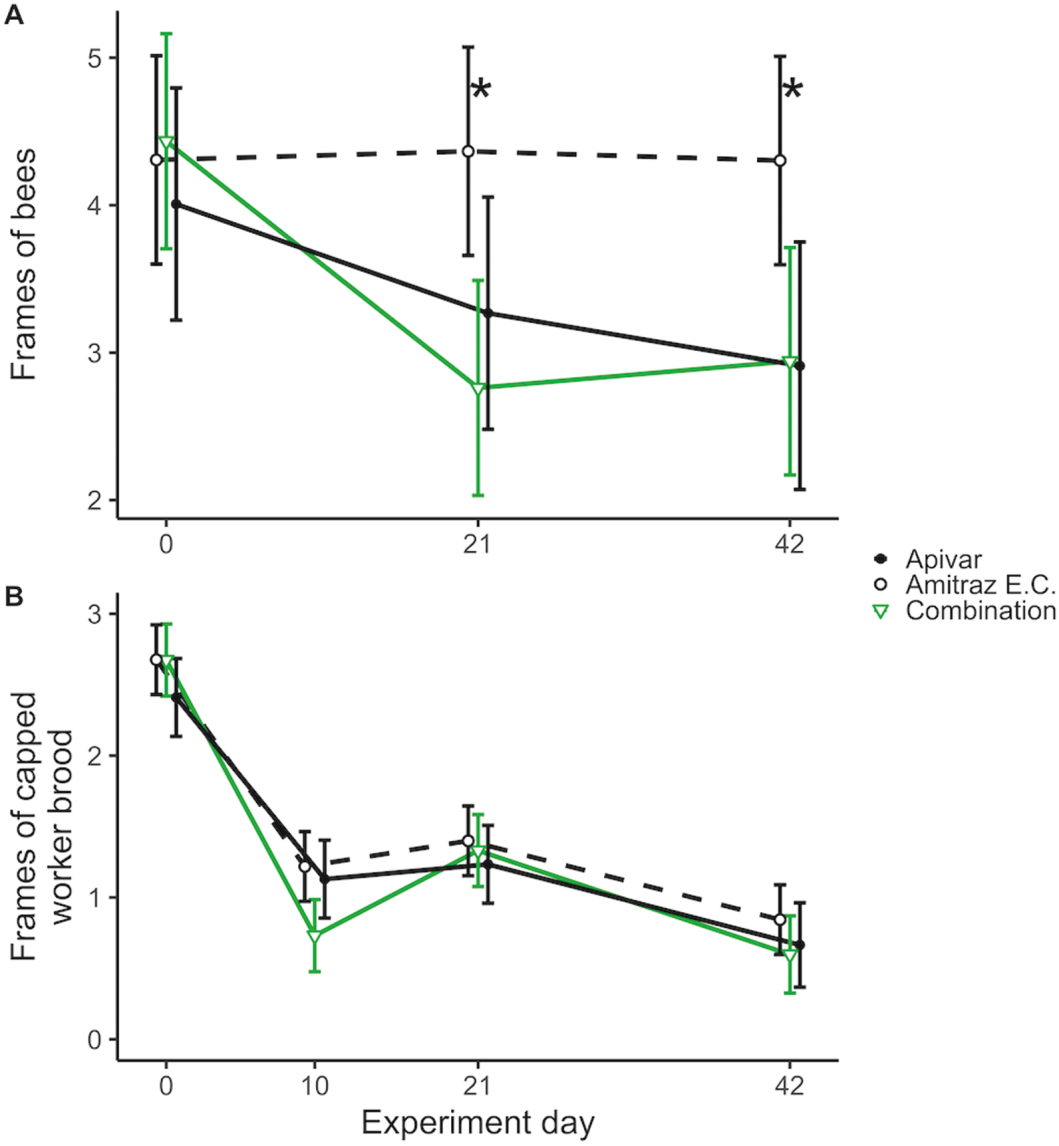
Strength of *Apis mellifera* honey bee colonies, as measured by: A) population of adult bees; B) population of developing worker bees in the capped stage (“capped worker brood”). Estimated marginal means within days are shown based on linear mixed models no. 4 and 6, respectively (Table 1), and error bars indicate 95% confidence intervals. Asterisks indicate one or more pairwise differences between treatments within that day significant at *P* < 0.05. Point estimates and 95% confidence intervals can be found in Table 2.

On day 0, we found no significant differences between the adult bee population in the Apivar, amitraz EC, and combination groups (*F*_2,71.27_ = 0.325, *P* = 0.735), with estimated populations of 4.0, 4.3, and 4.4 frames of bees, respectively (Fig. 2; estimates and 95% CIs in Table 2). By day 21, we observed significant differences between groups (*F* ratio_2,71.3_ = 5.192, *P* = 0.008). The amitraz EC group had 1.6 more frames of bees (95% CI: 0.4–2.8) than the combination group (*t*-ratio_71.3_ = −3.15, *P* = 0.007). However, we did not detect significant differences in bee population between the amitraz EC group and the Apivar group (*t*-ratio_71.3_ = −2.07, *P* = 0.104), nor between the Apivar group and the combination group (*t*-ratio_71.3_ = 0.94, *P* = 0.616). On day 42, there were significant differences between groups (*F* ratio_2,77.5_ = 4.52, *P* = 0.014). The amitraz EC group had 1.4 more frames of bees (95% CI: 0.1–2.7) than the Apivar group (*t*-ratio_77.5_ = −2.52, *P* = 0.036) and had 1.4 more frames of bees (95% CI: 0.1–2.6) than the combination group (*t*-ratio_76.6_ = −2.58, *P* = 0.031). The Apivar group was not significantly different from the combination group (*t*-ratio_80.2_ = −0.05, *P* = 0.998).

## Effects of Treatment on Capped Worker Brood Population

We found a significant effect of experiment day on frames of capped worker brood (χ^2^_3_ = 192.35, *P* < 0.001). Neither the effect of treatment, nor the interaction of treatment × experiment day on capped worker brood population were significant (χ^2^_2_ = 1.84, *P* = 0.399; and χ^2^_6_ = 10.77, *P* = 0.096, respectively), but because of our experiment designed to examine these effects, we retained these terms in the final model. When added to the a priori model, *Varroa* infestation rate of adult bees on day 0 was associated with lower capped worker brood population (χ^2^_1_ = 13.77, *P* < 0.001). However, the interaction of day 0 *Varroa* infestation rate × experiment day was not significant (χ^2^_3_ = 6.93, *P* = 0.074). Therefore, our final model included the effect of day 0 *Varroa* infestation rate, but omitted this interaction (Table 1). For each additional 1% infestation of adult bees, we found that colonies had 0.03 frames (95% CI: 0.015–0.045) less capped worker brood.

We generated point estimates for frames of capped worker brood for each treatment and day but did not perform post hoc tests within day because the interaction of treatment × experiment day on capped worker brood population was not significant. On day 0, colonies in the Apivar, amitraz EC, and combination groups had 2.4, 2.7, and 2.7 frames of capped worker brood, respectively (Fig. 2; estimates and 95% CIs in Table 2). On day 10, we estimated that the Apivar, amitraz EC, and combination groups had 1.1, 1.2, and 0.73 frames of capped worker brood, respectively. On day 10, we noted debris on the bottom boards from cannibalism of pupae in the combination group colonies, but we did not quantify this rate of cannibalism. The population of capped worker brood declined to the end of the trial, ending with approximately 0.7 frames of capped worker brood.

## Other Results

Of the 6 colonies which experienced queen loss during the trial, 4 colonies were in the Apivar group, and 2 colonies were in the combination group. As is normal during the fall, brood rearing declined over the course of the experiment (Table 2, Fig. 2).

## Discussion

*Varroa* infestations clearly harm colonies, and fall is a critical period during which high infestation rates are particularly damaging (Amdam et al. 2004). *Varroa* infestations had a substantial negative effect on adult bee population even during this short experiment, which complements the literature showing damage over longer time periods (e.g., Genersch et al. 2010) and underlines the need to control *Varroa* when they are at damaging levels. Colonies treated with amitraz EC ended the trial with the largest adult bee populations; relative to the amitraz EC group, colony populations were depressed in the Apivar group and in the combination group. In the context of high fall *Varroa* infestations, our results indicated that rapid control helps alleviate damage to colonies. Therefore, we suggest that registered treatments are needed that can provide rapid control. Our data also support the hypothesis that a combination treatment with Apivar (active ingredient, amitraz) and Apiguard (active ingredient, thymol) provides more rapid control of *Varroa* than Apivar alone. However, reductions in adult bee population indicated a tradeoff between control of *Varroa* and damage to colonies. This tradeoff needs to be optimized to exploit this combination of treatments for *Varroa* control.

Our omission of an untreated control group prevents us from comparing *Varroa* infestations and bee and brood populations of treated colonies against those of untreated colonies. If included, we would have expected negative controls to experience an increasing *Varroa* infestation as seen in other studies in fall when brood rearing is declining (Vandervalk et al. 2014)—and then to collapse rapidly given that the mean starting *Varroa* infestation of adult bees was near 10%, far exceeding the threshold.

### Effects of Amitraz EC Treatment

The results on day 21 of the experiment supported our initial assumption that amitraz EC provides rapid *Varroa* control, and that it does so without reducing the honey bee colony population. Our results validate the choice of amitraz EC as a positive control treatment whose effects we were trying to match with a combination treatment. We suspect that the rapid *Varroa* control by day 21 helped limit damage and enabled the colonies to maintain their adult bee population (Frey and Rosenkranz 2014). While *Varroa* on adult bees were nearly completely controlled by day 21, some *Varroa* remained in capped brood cells. We think these *Varroa* in the reproductive phase were the source of the apparent rebound in *Varroa* infestation rates from day 21 to day 42 after the last amitraz EC towel had been removed. This confirms that *Varroa* in capped brood cells are at least partly protected not only from organic nonvolatile treatments, but also from synthetic treatments (Lupo and Gerling 1987, Koeniger and Fuchs 1988).

### Effects of Apivar Treatment

While *Varroa* infestation rates decreased in Apivar-treated colonies, this decrease was slower than in amitraz EC-treated colonies. This is consistent with the view of Apivar as a treatment that provides extended rather than rapid control of *Varroa* infestations (Honey Bee Health Coalition 2021). We suspect that the continued high infestation rates (especially of the brood) on day 21 compromised the colony population by day 42 through continued damage from *Varroa* parasitism. This supports the idea that Apivar is best used when *Varroa* infestations are moderate (Honey Bee Health Coalition 2021), and that it is less useful when *Varroa* infestations have reached damaging levels like those at the beginning of this study. Nevertheless, the *Varroa* infestation rates in the Apivar group were not significantly different from the other groups on day 42.

At the end of the experiment, the colonies treated with Apivar had 1.4 fewer frames of bees than those treated with amitraz EC. This corresponds to a 32% lower colony population and an absolute deficit of approximately 3,350 bees (calculation based on Delaplane et al. 2013). This deficit in fall population is of a magnitude that has been predictive of winter mortality in other studies (Guzmán-Novoa et al. 2010), which indicates it is biologically significant, especially because the colonies were relatively weak to begin with. Furthermore, payment for pollination contracts is often determined based on “frames of bees” that count less than fully occupied frames (Goodrich 2019). Differences in colony population such as the ones we observed would be detectable during pollination audits and could be economically significant.

### Effects of Combined Apivar and Apiguard Treatment

The combined treatment with Apivar and Apiguard provided more rapid *Varroa* control than Apivar alone (considering the lower infestation rate of worker brood and the trend toward lower infestation rate of adult bees on day 21). However, colonies that received the combination treatment had 31% lower adult bee populations on day 42 than those that received amitraz EC. This is similar to the reduction seen in the Apivar-only group, and again, we think this reduction is biologically significant. This reduction in adult bee population may have resulted from a reduction in capped worker brood population shortly after application of the combination treatment. While our data on capped worker brood population provided only weak evidence for brood suppression by the combination treatment (nonsignificant interaction *P* value of 0.096), the estimated effect was substantial and is supported by corroborating evidence. Specifically, we estimated that the combination of Apivar and Apiguard caused a deficit of 0.49 frames of capped worker brood on day 10 relative to the amitraz EC group, which equates to a 40% reduction and a deficit of 3,277 capped worker brood cells (Delaplane et al. 2013). This estimated deficit of capped brood cells aligns closely with the estimated deficit of 3,305 adult bees on day 42 (Delaplane et al. 2013) and aligns with the visual evidence of brood cannibalism observed in this treatment group. Overall, this indicates that brood suppression occurred when combining Apiguard with Apivar, even though only one 51-ml application of Apiguard was used instead of the 2 sequential doses permitted by the label. We cannot explicitly determine to what extent the more rapid control of *Varroa* was due to cultural control (brood suppression), chemical control (direct efficacy against *Varroa*), or both. Also, because we omitted an untreated control, we can only compare the effects of the combination relative to the positive controls and because we omitted an Apiguard-only treatment, we are unable to disentangle direct effects of Apiguard from interactive effects of Apiguard plus Apivar treatment.

### Implications for Future Work

We think the brood suppressive effect of Apiguard (Floris et al. 2004) and other Varroacides could be considered both chemical and cultural control and our results provide preliminary evidence that suppressing brood intentionally with a treatment may be useful for *Varroa* control. However, brood suppression leads directly to reductions in adult bee population—whether caused by Varroacide applications or by interventions such as queen caging (Jack et al. 2020). This means a tradeoff exists between short-term reductions in bee population and improved control of *Varroa*. Future experiments testing a combination of Apivar and Apiguard should attempt to optimize the tradeoff between control of *Varroa* and effects on colony strength—which both influence survival over winter (Guzmán-Novoa et al. 2010). Because brood rearing rapidly decreased during the trial (which was expected at the time of year), there would have been limited opportunities for colonies to raise brood in the second half of the trial. If this were remedied, colony outcomes may have been better. We predict that earlier treatment could allow colonies more time post-treatment to raise brood, build up their adult populations and raise their final winter bees under lower *Varroa* infestation rates (Mattila et al. 2001) and perhaps avoid the deficit in colony population we observed. Note that earlier in the season, higher temperatures are likely, which increase the potential for treatments such as Apiguard to damage colonies. Therefore, it may be necessary to adjust treatment protocols, for example by using a lower dose of Apiguard as recommended by the label at high temperatures (US EPA 2018).

We think it is important for US honey bee researchers to include the unregistered amitraz EC treatment as a positive control when performing tests of alternative treatments against *Varroa*. As far as we are aware, our study and another study in this issue (Jack et al. 2024) are the first peer-reviewed US studies of treatments against *Varroa* since the late 1980s (e.g., Witherell and Herbert 1988) to include amitraz EC, despite it being widely used in the US beekeeping industry (Honey Bee Health Coalition 2021). By providing concrete data on its effects, we not only fill a gap in the literature, but also can communicate our findings on alternative treatments in a way that is more relevant to beekeepers who currently rely on amitraz EC. We suspect that beekeepers are more likely to adopt new or optimized registered treatments if they see how these compare against a treatment they have confidence in. Our research as well as that of others in this special issue (Jack et al. 2024) demonstrate that amitraz EC can provide higher efficacy or more rapid control than currently registered treatments. This makes it an especially relevant positive control—so that we can develop registered treatments that match or exceed the performance of amitraz EC. It is particularly urgent to provide beekeepers with alternative treatments, given that *Varroa* has started to develop resistance to amitraz (Rinkevich 2020, Hernández-Rodríguez et al. 2022). By investigating practices that allow combination of active ingredients, or enable rotation with more effective non-amitraz treatments, researchers could help US beekeepers be better prepared for a scenario in which *Varroa* mites develop extensive and severe resistance to amitraz, as has occurred with other Varroacides (Jack and Ellis 2021).

## Supplementary Material

ieae022_suppl_Supplementary_Figures_S1

## Data Availability

Data and analysis code are available on GitHub: https://doi.org/10.5281/zenodo.10612431 (Aurell et al. 2024b).
