# Supplementary material for: Combined treatment with amitraz and thymol to manage Varroa destructor mites (Acari: Varroidae) in Apis mellifera honey bee colonies (Hymenoptera: Apidae)
Source: J Insect Sci. 2024 May 28;24(3):12. doi: 10.1093/jisesa/ieae022 (PMC11132131; doi:10.1093/jisesa/ieae022)
Supplement: ieae022_suppl_Supplementary_Figures_S1 [file ieae022_suppl_supplementary_figures_s1.pdf]

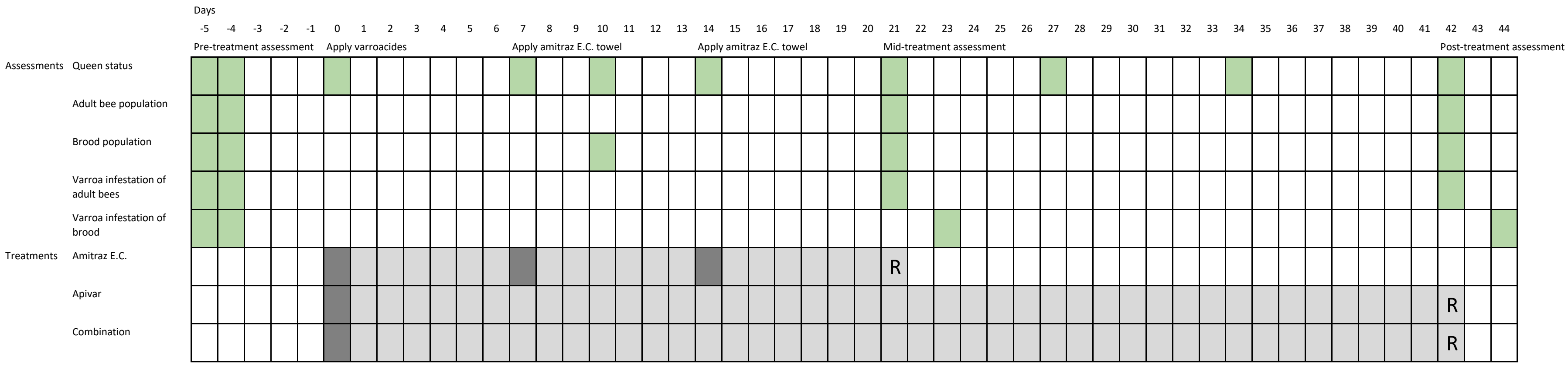

Supp Fig. S1. Schedule of assessments and treatments before, during, and after the 42-day experimental period. For assessments, a green-shaded square indicates the day a type of assessment was performed. For treatments, a dark grey-shaded square indicates a treatment was applied, a light grey-shaded square indicates that treatments were present, and “R” indicates that treatments were removed.
